# ADAM17-mediated EGFR ligand shedding directs macrophage-promoted cancer cell invasion

**DOI:** 10.1172/jci.insight.155296

**Published:** 2022-09-22

**Authors:** Sebastian P. Gnosa, Laia Puig Blasco, Krzysztof B. Piotrowski, Marie L. Freiberg, Simonas Savickas, Daniel H. Madsen, Ulrich auf dem Keller, Pauliina Kronqvist, Marie Kveiborg

**Affiliations:** 1 Biotech Research and Innovation Centre (BRIC), University of Copenhagen, Copenhagen, Denmark.; 2 Department of Biotechnology and Biomedicine, Technical University of Denmark, Copenhagen, Denmark.; 3 National Center for Cancer Immune Therapy (CCIT-DK), Department of Oncology, Copenhagen University Hospital, Herlev, Denmark.; 4 Institute of Biomedicine and; 5 Department of Pathology, University of Turku, Turku, Finland.

**Keywords:** Oncology, Cancer, Macrophages, Proteases

## Abstract

Macrophages in the tumor microenvironment have a substantial impact on tumor progression. Depending on the signaling environment in the tumor, macrophages can either support or constrain tumor progression. It is therefore of therapeutic interest to identify the tumor-derived factors that control macrophage education. With this aim, we correlated the expression of A Disintegrin and Metalloproteinase (ADAM) proteases, which are key mediators of cell-cell signaling, to the expression of protumorigenic macrophage markers in human cancer cohorts. We identified ADAM17, a sheddase upregulated in many cancer types, as a protein of interest. Depletion of ADAM17 in cancer cell lines reduced the expression of several protumorigenic markers in neighboring macrophages in vitro as well as in mouse models. Moreover, *ADAM17^–/–^* educated macrophages demonstrated a reduced ability to induce cancer cell invasion. Using mass spectrometry–based proteomics and ELISA, we identified heparin-binding EGF (HB-EGF) and amphiregulin, shed by ADAM17 in the cancer cells, as the implicated molecular mediators of macrophage education. Additionally, RNA-Seq and ELISA experiments revealed that ADAM17-dependent HB-EGF ligand release induced the expression and secretion of CXCL chemokines in macrophages, which in turn stimulated cancer cell invasion. In conclusion, we provide evidence that ADAM17 mediates a paracrine EGFR-ligand-chemokine feedback loop, whereby cancer cells hijack macrophages to promote tumor progression.

## Graphical abstract

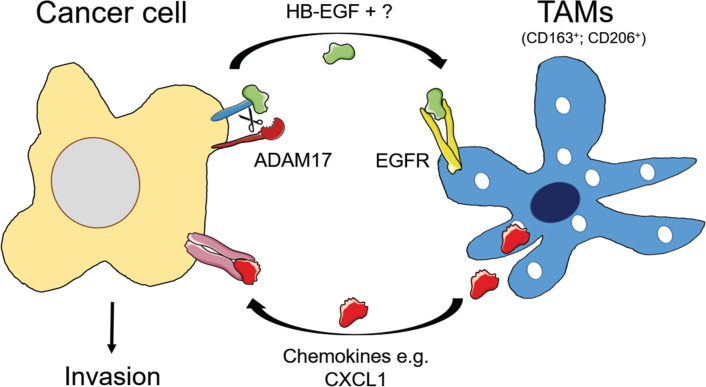

## Introduction

Cancer development and metastasis depend greatly on the interaction of cancer cells with the environment, including macrophages, which infiltrate tumors in high numbers and often indicate a poor prognosis (1, 2). Macrophages are specialized cells that continuously patrol and monitor the body to resolve infections and clear dying cells. When abnormalities are detected, for example during wound healing, macrophages eliminate intruding microorganisms, orchestrate the immune system, promote and resolve inflammation, and support cell proliferation and tissue remodeling (3). Factors in the microenvironment drive the education of the macrophages toward specialized cell states, with 2 extreme states described as a proinflammatory, classically activated M1 state and an antiinflammatory, alternatively activated M2 state (4). However, multiple studies reveal that macrophages exist in a continuum of cell states and functions and that they oscillate between different activation states (5). Also in tumors, macrophages are diverse in their phenotypes and either support or suppress tumor progression. Tumor-associated macrophages (TAMs) initially attempt to restore a normal structure in tumors, analogous to classically M1 activated macrophages (6). However, secretion and proteolytic release of certain cytokines and growth factors, such as colony stimulating factor-1 (CSF-1) (7) and interleukin-4 (IL-4) (8), by the tumor cells educate TAMs toward a tumor-promoting phenotype, sharing many features of alternatively activated M2 macrophages. Thereby, TAMs can support tumor growth, metastasis, and immune evasion and protect tumor cells from chemotherapy (9–11). The TAM phenotype, being pro- or antitumorigenic, depends on the origin of the tumor and on the exact signaling within the tumor microenvironment (TME). While attempts to deplete TAMs have not shown any pronounced therapeutic effects, reeducation toward an antitumor phenotype offers an alternative strategy (12). Thus, finding the signals released by cancer cells to educate macrophages and identifying the mechanisms of their release are crucial for the development of future targeting approaches.

Many key signaling molecules are shed from the cell surface by A Disintegrin and Metalloproteinase (ADAM), placing these enzymes as central regulators of cell-cell communication (13). ADAMs are a family of 21 membrane-anchored metalloproteinases, whereby 13 have a functional protease domain. Some of these, including ADAM9, ADAM10, ADAM12, ADAM15, and ADAM17, are shown to be upregulated in both tumor tissues and cancer cell lines, and their expression correlates to adverse patient survival and/or treatment response (14–20). Despite multiple reports showing the importance of ADAM proteases in cancer cell proliferation, adhesion, migration, and invasion (16, 21, 22), there are no studies evaluating the function of ADAMs on regulating the polarization of TAMs.

Here, we report that the expression of ADAM17 correlates to the expression of protumorigenic macrophage markers in human cohorts and regulates the phenotype of macrophages in mouse tumors. We show that ADAM17 sheds heparin-binding EGF (HB-EGF) and amphiregulin (AREG) from the cancer cell surface, leading to EGFR activation in macrophages. Moreover, we demonstrate that ADAM17/HB-EGF signaling regulates the education of protumorigenic macrophages, the expression and secretion of chemokines, as well as macrophage-induced cancer cell dissemination. Our study sheds light on the interplay between cancer cells and their environment and in particular the role of ADAM17 controlling the cellular identity of tumor-resident macrophages and their function.

## Results

### ADAM17 expression correlates to the expression of protumorigenic macrophage markers in multiple cancer types.

**Figure 1:**
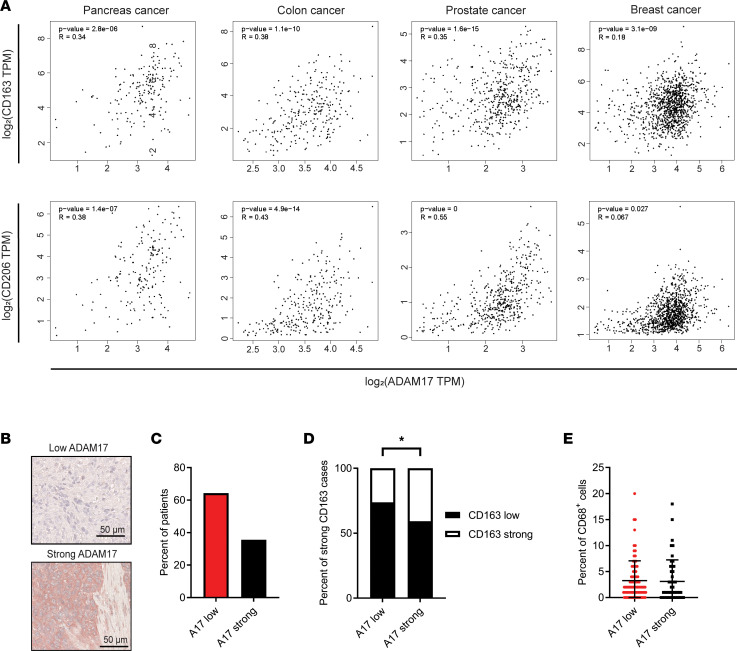
ADAM17 expression correlates to protumorigenic macrophage markers CD163 and CD206 in human cancer. (**A**) Correlations between mRNA expression of ADAM17 and expression of CD163 (top) and CD206 (bottom) in pancreas, colon, prostate, and breast cancer obtained from TCGA database and analyzed by the GEPIA tool. TPM, transcripts per million. (**B**) Representative IHC images of low and strong ADAM17 staining in a triple-negative breast cancer cohort (*n* = 159). (**C**) Percentage of low and strong ADAM17 (A17) staining within the cohort. (**D**) Percentage of strong CD163^+^ cases with low or strong ADAM17 staining. (**E**) Percentage of CD68-positive cells in cases with low or strong ADAM17 staining. Mean and standard deviation indicated. Pearson’s correlation for **A** and χ^2^ test for **C**–**E** were applied to test for significance; *P ≤ 0.05.

To assess the role of ADAMs in macrophage education, we initially asked whether the expression of catalytically active ADAMs correlates to the expression of protumorigenic macrophage markers in human cancer. For this, we used the online Gene Expression Profiling Interactive Analysis (GEPIA) tool (23) to analyze transcriptome data available from The Cancer Genome Atlas (TCGA) database. We found a positive correlation between the expression of ADAM17 and the protumorigenic macrophage markers CD163 and CD206 in several types of solid tumors (Figure 1A).

To further evaluate the correlation between ADAM17 and the protumorigenic macrophage markers, we stained a triple-negative breast cancer cohort, including tumor tissue from 159 patients, for ADAM17 by immunohistochemistry (IHC) (Figure 1B). This cohort has previously been analyzed for the expression of the protumorigenic macrophage marker CD163 and the general macrophage marker CD68 by IHC (24). In 35.6% of the samples, we detected a strong staining of ADAM17 in the tumor (Figure 1C), confirming previous reports of high ADAM17 expression in triple-negative breast cancer (25). Moreover, we found a positive correlation when comparing the stainings for ADAM17 and CD163 (Figure 1D), supporting the strong correlation observed at the RNA level. This is very interesting, given the fact that the activity of ADAM17 is controlled in many ways (26). The percentage of CD68^+^ macrophages, however, did not differ between tumors with low and strong ADAM17 staining (Figure 1E).

Together, our findings demonstrate that while ADAM17 expression does not correlate to overall macrophage numbers, it is positively associated with the expression of CD206 and/or CD163 — markers of protumorigenic macrophages in human tumors.

### ADAM17 is required for protumorigenic macrophage education.

**Figure 2:**
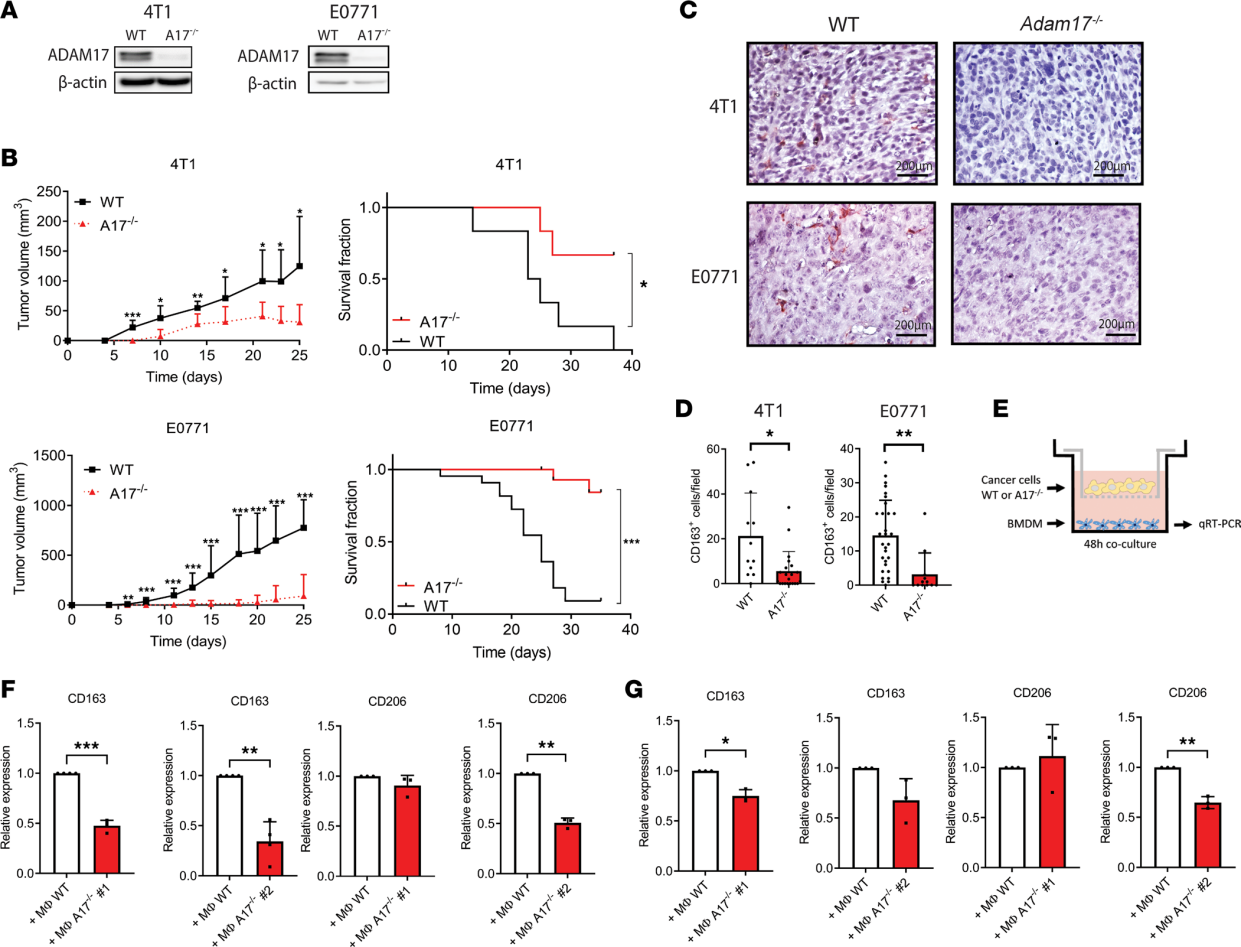
ADAM17 is required for protumorigenic macrophage education. (**A**) Western blot of ADAM17 protein expression in WT and *Adam17^–/–^* (A17^–/–^) 4T1 and E0771 cell lines (representative of 3 repeats). β-Actin was used as control. (**B**) *Left*: Average tumor volume (mm^3^) ± standard deviation; *Right*: Survival curves of WT or *Adam17^–/–^* 4T1 (clone 2, *n* = 6 mice per group, *top*) and E0771 (clone 1, *n* = 22 mice per group, *bottom*) cells injected into the mammary fat pad of BALB/c or C57BL/6JRj mice, respectively. (**C**) Representative IHC stainings for CD163 in WT or *Adam17^–/–^* 4T1 and E0771 tumors. Scale bar: 200 μm. (**D**) Quantified CD163-positive cells/field from 3 fields/tumor in WT and *ADAM17^–/–^* 4T1 (*n* = 4 and 7, respectively) and E0771 (*n* = 10 and 4, respectively) tumors. (**E**) Experimental setup for qRT-PCR of bone marrow–derived macrophages (BMDM) upon coculture with cancer cells (used in **F** and **G**). (**F**) Relative *CD163* (*n* = 4) and *CD206* (*n* = 3) mRNA expression in macrophages cocultured with WT or 2 clones of *Adam17^–/–^* 4T1 cells, determined by qRT-PCR. β_2_ microglobulin (B2M) was used as control. (**G**) Relative *CD163* (*n* = 3) and *CD206* (*n* = 3) mRNA expression in macrophages cocultured with WT or 2 clones of *Adam17^–/–^* E0771 cells, determined by qRT-PCR. B2M was used as control. Mean and standard deviation indicated. Two-sided, unpaired Student’s *t* test (**B**, **D**, **F**, and **G**) and log-rank (**B**) tests were applied to test for significance; *P ≤ 0.05, **P ≤ 0.01, ***P ≤ 0.001.

We next examined whether ADAM17 directly regulates the number of CD163^+^ cells, using orthotopic mouse mammary tumors. Using CRISPR/Cas9 gene editing, we knocked out *Adam17* expression (*Adam17*^–/–^) in the mouse breast cancer cell lines 4T1 and E0771 (Figure 2A). We injected these *Adam17*^‑/–^ cells and corresponding nonedited parental wild-type (WT) cells into the mammary fat pad of female WT mice. Interestingly, ADAM17 depletion significantly decreased tumor growth and increased mouse survival in both breast cancer cell lines (Figure 2B). We observed no significant difference in proliferation between WT and *Adam17^–/–^* cells in vitro in either 4T1 or E0771 cell lines (Supplemental Figure 1, A and B; supplemental material available online with this article; https://doi.org/10.1172/jci.insight.155296DS1), indicating that cell-autonomous changes in cancer cell proliferation do not explain the delay in tumor growth seen in vivo.

We then stained the tumors for CD163 by IHC. Demonstrating that ADAM17 regulates the education of macrophages, we found significantly fewer CD163^+^ cells in *Adam17*^–/–^ tumors of both cell lines (Figure 2, C and D). Moreover, FACS analysis revealed that the number of CD11b^+^F4/80^+^ macrophages was similar in 4T1 WT and *Adam17*^‑/–^ tumors (data not shown).

Given that ADAM17 sheds many membrane-anchored signaling molecules and receptors from the cell surface (27), we asked whether ADAM17-dependent macrophage education is due to ADAM17-mediated shedding of 1 or more signaling factors. To test this, we isolated bone marrow–derived macrophages (BMDMs) from female WT mice and cultured them with WT or *Adam17*^–/–^ cancer cells, separated by a porous membrane allowing only soluble molecules to pass. After 48 hours, we analyzed the expression of protumorigenic macrophage markers in BMDMs using quantitative real-time PCR (qRT-PCR) (Figure 2E). In macrophages cocultured with *Adam17*^–/–^ 4T1 (Figure 2F) or E0771 (Figure 2G) breast cancer cells, we found significantly lower expression of *CD163* and/or *CD206* in at least 1 of 2 *Adam17*^–/–^ clones, as compared with macrophages cocultured with WT cancer cells. Moreover, we found that macrophages polarized by ADAM17-deficient cancer cells showed a tendency toward decreased expression of several other protumorigenic markers (Supplemental Figure 2, A and B).

Together, these data indicate a central role of ADAM17 in the education of protumorigenic macrophages.

### ADAM17 drives macrophage-induced invasion.

**Figure 3:**
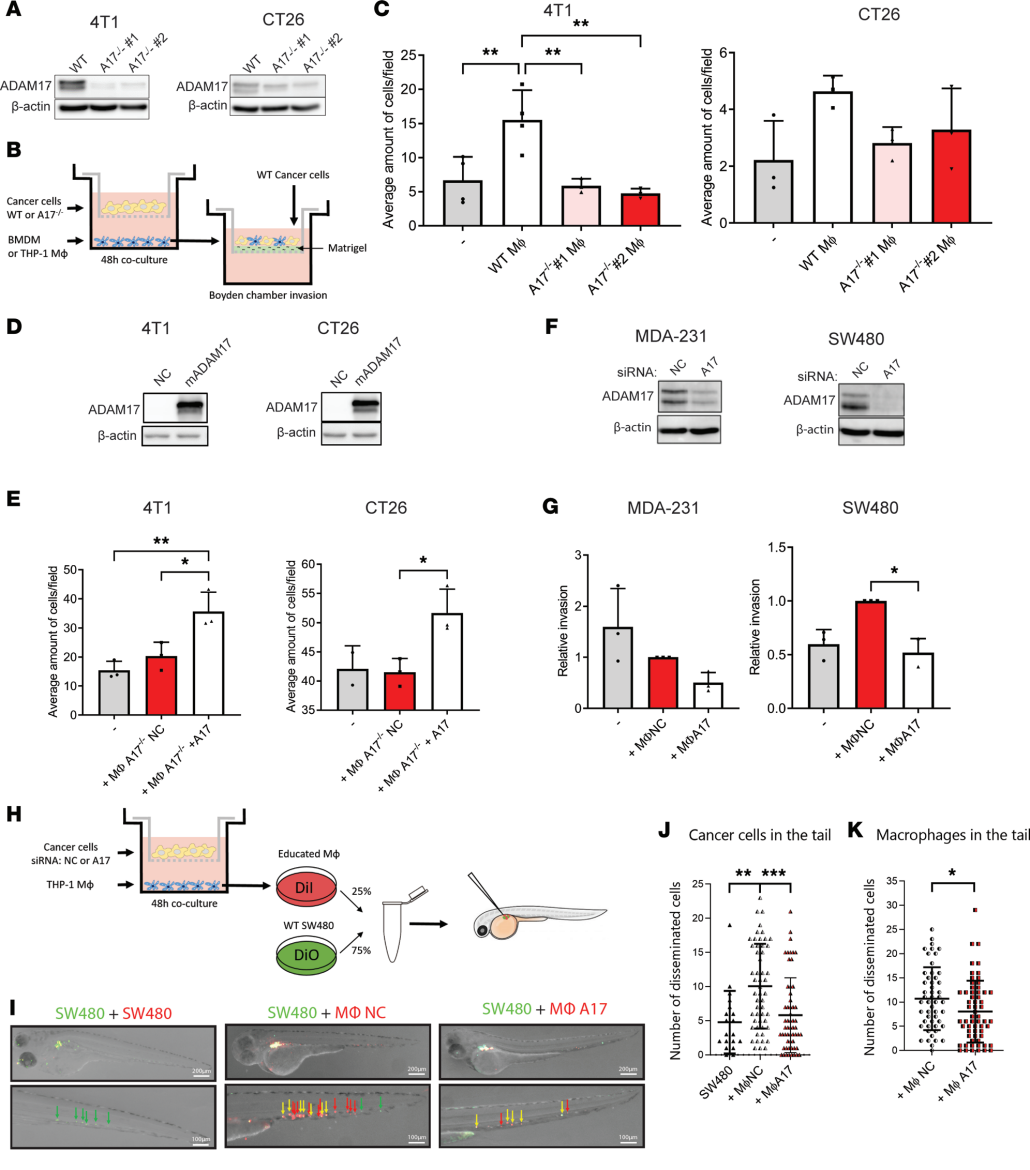
Cancer cells educate macrophages toward an invasion-promoting phenotype via ADAM17-dependent soluble factors. (**A**) Western blot of ADAM17 WT and *Adam17^–/–^* (A17^–/–^) clones 1 and 2 4T1 and CT26 cell lines (representative of 3 repeats). β-Actin served as control. (**B**) Experimental setup for **C**, **E**, and **G**. (**C**) Average invaded cells/field of WT 4T1 and CT26 cells alone or with BMDMs educated with WT or *Adam17^–/–^* cells (*n* = 3). (**D**) ADAM17 Western blot of *Adam17^–/–^* 4T1 and CT26 cell lines expressing empty vector (negative control, NC) or mouse ADAM17 (mADAM17) (representative of 3 repeats). β-Actin served as control. (**E**) Invaded cells/field of WT 4T1 or CT26 cell lines alone or with BMDMs educated with *Adam17^–/–^* NC or mADAM17 cancer cells (*n* = 3). (**F**) ADAM17 Western blot of MDA-231 and SW480 cells transfected with NC or ADAM17 (A17) siRNA (representative of 3 repeats). β-Actin served as control. (**G**) Invaded cells/field of WT cell lines with THP-1-derived macrophages (THP-1MΦ) educated by NC or ADAM17 siRNA–transfected MDA-231 and SW480 cells (*n* = 3). (**H**) Experimental setup for the zebrafish embryo dissemination assay in **I**–**K**. (**I**) Example of tail foci in embryonic zebrafish injected with SW480 cells alone or with THP-1MΦ educated with NC or A17 siRNA–transfected SW80 cells. Arrows: green: cancer cells, red: macrophages, yellow: both cancer cells and macrophages. Scale bar: 200 μm (top), 100 μm (bottom). Quantification of cancer cell (**J**) and macrophage (**K**) foci in tail regions 24 hours after injection with SW480 cells alone (*n* = 19) or with THP-1MΦ educated with NC siRNA–transfected SW80 cells (*n* = 53) or A17 siRNA–transfected SW80 cells (*n* = 57). Mean and standard deviation indicated. Data in **C**, **E**, and **J** were analyzed by 1-way ANOVA with Dunnett’s multiple comparison test, and data in **G** were analyzed by Kruskal-Wallis with Dunn’s multiple comparison test. Data in **K** were analyzed using unpaired 2-sided Student’s *t* test. *P ≤ 0.05, **P ≤ 0.01, ***P ≤ 0.001.

A hallmark of TAMs is to support tumor cell invasion (1). So, we asked whether macrophages polarized by cancer cells promote cancer cell invasion and whether that relies on ADAM17-mediated shedding of 1 or more factors from the cancer cells. To test this, WT or *Adam17*^–/–^ cancer cells (breast 4T1 and colon CT26, Figure 3A) were cultured with BMDMs for 48 hours, separated by a porous membrane. Macrophages were subsequently seeded together with WT cancer cells labeled with 1,1’-dioctadecyl3,3,3’3’-tetramethylindocarbocyanine (DiI) in Matrigel-coated Boyden chambers, to evaluate the effects on cancer cell invasion (Figure 3B). Although reaching statistical significance in 4T1 cells only, these experiments revealed that ADAM17 is involved in macrophage-induced cancer cell invasion and that 1 or more ADAM17-dependent soluble factors regulates this process (Figure 3C). These findings were confirmed in the mouse breast cancer cell line E0771 and the mouse colon cancer cell line MC38 (Supplemental Figure 3, A and B). To further validate our findings, we used the sleeping beauty transposon technology to reexpress ADAM17 in the 4T1 and CT26 *Adam17*^–/–^ cell lines (Figure 3D). Indeed, macrophages cocultured with ADAM17-reexpressing cells regained the ability to induce cancer cell invasion (Figure 3E).

In order to extend the study of ADAM17 in the crosstalk between macrophages and cancer cell invasion to an in vivo context, we took advantage of the zebrafish embryo dissemination model (28, 29). Due to a high number of macrophages needed, we used the THP-1 macrophage cell line. First, we evaluated whether we could confirm the role of cancer cell–derived ADAM17 expression on macrophage-induced cancer cell invasion in this model. For that evaluation, we cocultured PMA-differentiated THP-1 cells with MDA-MB-231 (MDA-231) human breast cancer cells or SW480 human colon cancer cells, treated with either negative control (NC) or ADAM17 siRNA for 48 hours (Figure 3F), and evaluated the macrophage potential to induce cancer cell invasion (Figure 3B). MDA-231–polarized THP-1 macrophages were not able to induce cancer cell invasion (Figure 3G), possibly due to the already highly invasive potential of these cells. However, THP-1 macrophages polarized by ADAM17-deficient MDA-231 cells reduced the invasion of WT MDA-231 cells. Confirming our BMDM data, THP-1 macrophages polarized by WT SW480 cells clearly induced the invasion of cocultured WT cancer cells, and when THP-1 macrophages were polarized by ADAM17-deficient SW480 cancer cells, the increase in cancer cell invasion was completely lost (Figure 3G).

Based on these findings, we then used the SW480 cells in the zebrafish embryo invasion model. We cocultured PMA-induced THP-1 macrophages with SW480 cancer cells, transfected with either NC or ADAM17 siRNA for a total of 48 hours. The activated macrophages were then labeled with DiI, mixed with untreated SW480 cells labeled with 3-octadecyl-2-[3-(3-octadecyl-2(3H)-benzoxazolylidene)-1-propenyl]-, perchlorate (DiO), in a 1:4 ratio, and injected into the perivitelline space of WT zebrafish. As a control, DiI-labeled SW480 cells were mixed with DiO-labeled SW480 cells in the same ratio (Figure 3H). Cancer cell and macrophage dissemination through intravasation to the tail region was monitored 24 hours after cell injection (Figure 3I). The zebrafish in vivo invasion model revealed that injection of SW480 cancer cells with preconditioned macrophages enhanced cell dissemination into the tail region and that cancer cell–derived ADAM17 was required to obtain this effect (Figure 3J). Interestingly, the number of disseminated macrophages was reduced when the cells were primed by ADAM17-deficient cancer cells (Figure 3K).

Taken together, cancer cells educate macrophages toward a protumorigenic phenotype, promoting the invasion of cancer cells via ADAM17-dependent soluble factor(s).

### ADAM17-mediated EGFR ligand shedding promotes macrophage education and macrophage-induced cancer cell invasion.

**Figure 4:**
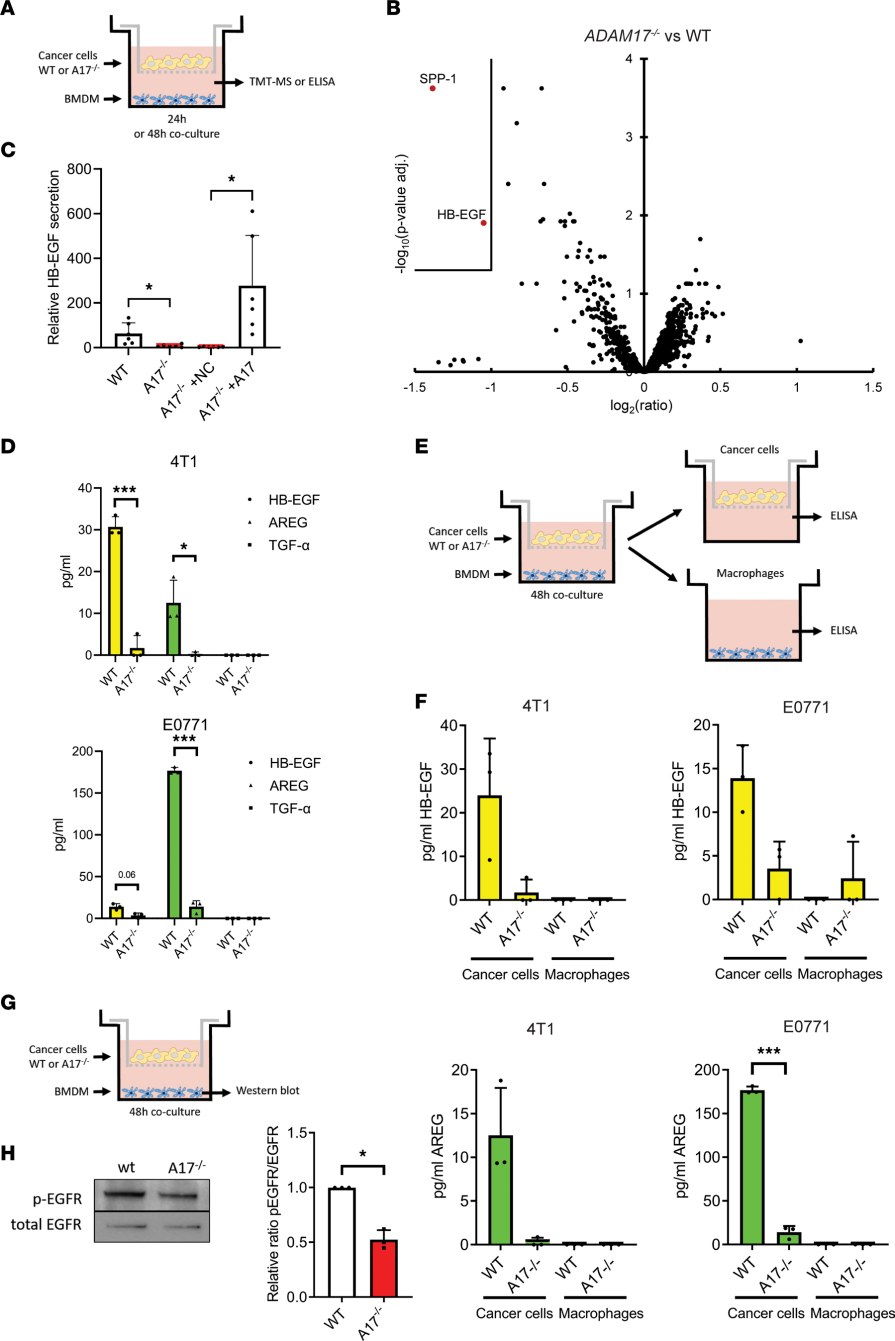
Soluble EGFR ligands are decreased in *Adam17^–/–^* cocultures. (**A**) Experimental setup for secretome analyses by TMT-MS/MS and ELISA of BMDMs cocultured with cancer cells (used in **B**–**D**). (**B**) Volcano plot of proteins identified in secretomes of WT or *Adam17^–/–^* (A17^–/–^) 4T1 cancer cell-BMDM cocultures by TMT-MS/MS. Significantly altered proteins are shown in red. SPP-1, secreted phosphoprotein-1. (**C**) Relative HB-EGF secretion in WT, *Adam17^–/–^*, and *Adam17^–/–^* expressing empty vector (NC) or ADAM17 4T1 cells, determined by parallel reaction monitoring–targeted (PRM-targeted) MS. (**D**) Secretion of HB-EGF, AREG, and TGF-α in WT or *Adam17^–/–^* 4T1 (*top*) and E0771 (*bottom*) cancer cell-BMDM cocultures, determined by ELISA (*n* = 3). (**E**) Experimental setup for HB-EGF and AREG ELISA of BMDM-cancer cell coculture media. (**F**) HB-EGF and AREG ELISA of WT or *Adam17^–/–^* 4T1 and E0771 cell lines and BMDM media, 16 hours after coculture (*n* = 3). (**G**) Experimental setup for Western blot of BMDMs upon cancer cell coculture. (**H**) Western blot of phosphorylated EGFR (p-EGFR) (Tyr1068) and total EGFR in BMDMs cocultured with either WT or *Adam17^–/–^* 4T1 cells, quantified as p-EGFR/total EGFR (*n* = 3). Mean and standard deviation indicated. Data in **C** were analyzed using Welch ANOVA with correction for multiple comparisons by controlling FDR using Benjamini-Hochberg method, data in **D** were analyzed using 2-way ANOVA with Holm-Šidák correction for multiple analysis, data in **F** were analyzed by 1-way ANOVA with Dunnett’s multiple comparison test, and data in **H** were analyzed using 2-sided, unpaired Student’s *t* test, *P ≤ 0.05, ***P ≤ 0.001.

There are currently over 80 ADAM17 substrates reported, including cytokines, growth factors, and their receptors (30). To identify the factor(s) responsible for ADAM17-dependent macrophage education, we performed an unbiased quantitative tandem mass tag–mass spectrometry–based (TMT-MS–based) proteomics analysis of the secretome from macrophages cocultured with WT or *Adam17*^–/–^ 4T1 cancer cells (Figure 4A). For statistically robust comparison of the groups, we exploited the multiplexing capabilities (10-plex) of TMT and analyzed 3 replicates per condition. We detected 7,921 unique peptides and 2,563 proteins (the raw data can be found in Supplemental Data 1), and of these, the HB-EGF was significantly downregulated in *Adam17*^–/–^ cocultures (Figure 4B). Given that ADAM17 is depleted in the cancer cell population, we speculated that the cancer cells are the source of HB-EGF, and we confirmed that reexpression of ADAM17 in *Adam17*^–/–^ 4T1 cells rescued the secretion of HB-EGF (Figure 4C). Since ADAM17 is known to shed multiple EGFR ligands, including HB-EGF, AREG, and transforming growth factor–α (TGF-α) (31), we also evaluated the shedding of these factors. Using ELISA, we found that TGF-α levels were below detection limit, while HB-EGF and AREG levels were both decreased in 4T1 and E0771 *Adam17*^–/–^ cocultures (Figure 4D).

To confirm cancer cells as the major source of HB-EGF and AREG release, we cocultured WT and *Adam17*^–/–^ 4T1 and E0771 cells with BMDMs for 48 hours. Subsequently, we separated the 2 cell types, collected the medium for another 16 hours, and measured HB-EGF and AREG levels by ELISA (Figure 4E). We found that both HB-EGF and AREG were secreted by the cancer cells but not by the macrophages (Figure 4F). We then asked whether the decreased release of EGFR ligands in *Adam17*^–/–^ cocultures affects the activation of EGFR in the macrophage population (Figure 4G). Western blot analysis revealed significantly lower EGFR phosphorylation at Tyr1068 in macrophages when cocultured with *Adam17*^–/–^ cells (Figure 4H).

**Figure 5:**
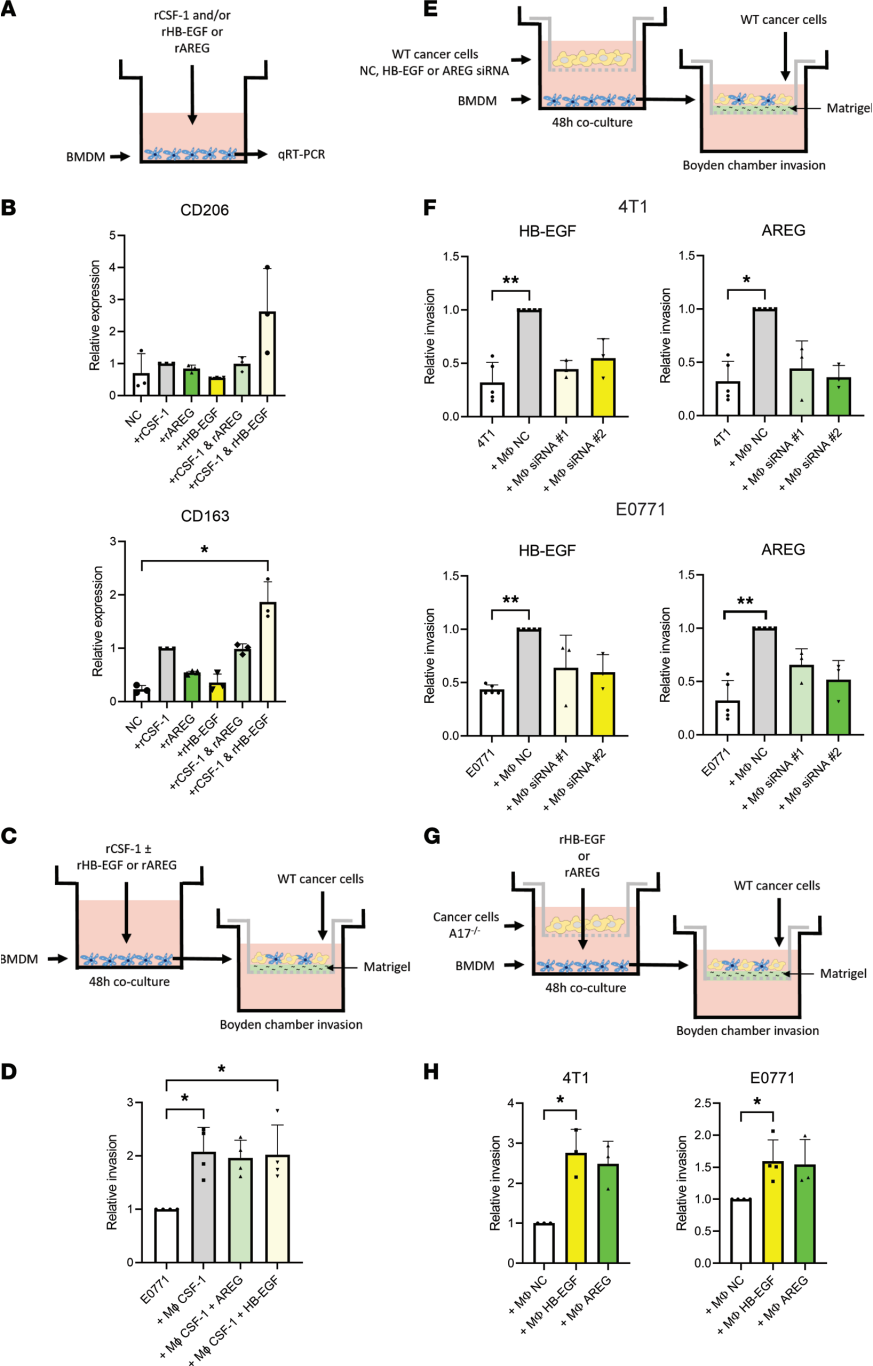
ADAM17-mediated EGFR ligand shedding promotes macrophage-induced cancer cell invasion. (**A**) Experimental setup for qRT-PCR of BMDMs treated with solvent (NC); recombinant CSF-1 (rCSF-1), HB-EGF (rHB-EGF), or amphiregulin (rAREG); or the combination of rCSF-1 and rHB-EGF or rAREG. (**B**) Relative CD163 and CD206 expression in BMDMs treated with NC, rCSF-1, rHB-EGF, rAREG, or the combination of rCSF-1 and rHB-EGF or rAREG (*n* = 3). (**C**) Experimental setup for Boyden chamber invasion assays of WT cancer cells together with BMDMs treated with rCSF-1, rCSF-1+rHB-EGF, or rCSF-1+rAREG. (**D**) Relative invasion of E0771 cells together with BMDMs treated with rCSF-1, rCSF-1+rHB-EGF, or rCSF-1+rAREG (*n* = 4). (**E**) Experimental setup for Boyden chamber invasion assays of WT cancer cells with BMDMs. BMDMs were cocultured with WT cancer cells transfected with nontargeting control (NC), HB-EGF, or AREG siRNA. (**F**) Relative number of invaded cells/field of WT 4T1 (*top*) and E0771 (*bottom*) cells alone or with BMDMs educated with NC, HB-EGF, or AREG siRNA–treated 4T1 or E0771 cells (*n* = 4). (**G**) Experimental setup for Boyden chamber invasion assays of WT cancer cells with BMDMs. BMDMs were cocultured with *Adam17^–/–^* (A17^–/–^) cancer cells with or without rHB-EGF or rAREG. (**H**) Relative invasion of 4T1 and E0771 cells with BMDMs educated by coculture with *Adam17^–/–^* cells in NC-, rHB-EGF–, or rAREG-containing medium (*n* = 3). Mean and standard deviation indicated. Data in **B**, **F**, **D**, and **H** were analyzed by Kruskal-Wallis with Dunn’s multiple comparison test, *P ≤ 0.05, **P ≤ 0.01.

Next, we evaluated whether EGFR ligands can directly polarize macrophages toward a protumorigenic phenotype (Figure 5A). Treating BMDMs with rHB-EGF or rAREG alone had no effect on the expression of CD163 or CD206, while rCSF-1 increased the expression of CD163 (Figure 5B). Interestingly, treating BMDMs with rCSF-1 together with rHB-EGF, but not rAREG, led to a tendency toward increased expression of the protumorigenic macrophage markers CD163 and CD206, as compared with CSF-1 alone (Figure 5B).

We then asked whether treatment of macrophages with rHB-EGF or rAREG together with rCSF‑1 would be enough to induce an invasion-promoting phenotype (Figure 5C). While macrophages treated with AREG or HB-EGF without CSF-1 did not survive the treatment period, macrophages treated with rCSF-1 alone induced cancer cell invasion, and adding rHB-EGF or rAREG did not further enhance this effect (Figure 5D). Given that the secreted level of CSF-1 was unchanged in the cocultures upon ADAM17 depletion, as shown by MS (Supplemental Data 2), we speculate that the polarization of macrophages toward an invasion-promoting phenotype occurs in response to a certain “signal amplitude,” which can be reached by combined CSF-1/EGFR signaling or by CSF-1 alone if the concentration is sufficiently high.

Next, we wanted to identify whether the reduced EGFR ligand release is responsible for the decreased ability of macrophages to support cancer cell invasion when cocultured with *Adam17*^–/–^ cancer cells. For that, we inhibited the expression of HB-EGF or AREG in the 4T1 and E0771 cells using 2 siRNAs (Supplemental Figure 4), cocultured these cells with BMDMs, and evaluated the macrophage-induced invasion of WT cancer cells, using the Boyden chamber assay (Figure 5E). Macrophages educated with either HB-EGF– or AREG-knockdown cancer cells were not able to induce cancer cell invasion in either 4T1 or E0771 cells, indicating that in the cocultures amplified EGFR signaling is required (Figure 5F). We then asked whether the addition of rHB-EGF or rAREG to *Adam17*^–/–^ cancer cell-macrophage cocultures could rescue the invasion-supporting macrophage phenotype (Figure 5G). Indeed, both rHB‑EGF and rAREG rescued the macrophage-induced cancer cell invasion in the 4T1 and E0771 breast cancer cell lines (Figure 5H).

Collectively, these data indicate that ADAM17-dependent HB-EGF and AREG release elicits an amplifying signal, required for the education of macrophages toward an invasion-supporting phenotype.

### Macrophage-derived CXCL1 induces cancer cell invasion.

**Figure 6:**
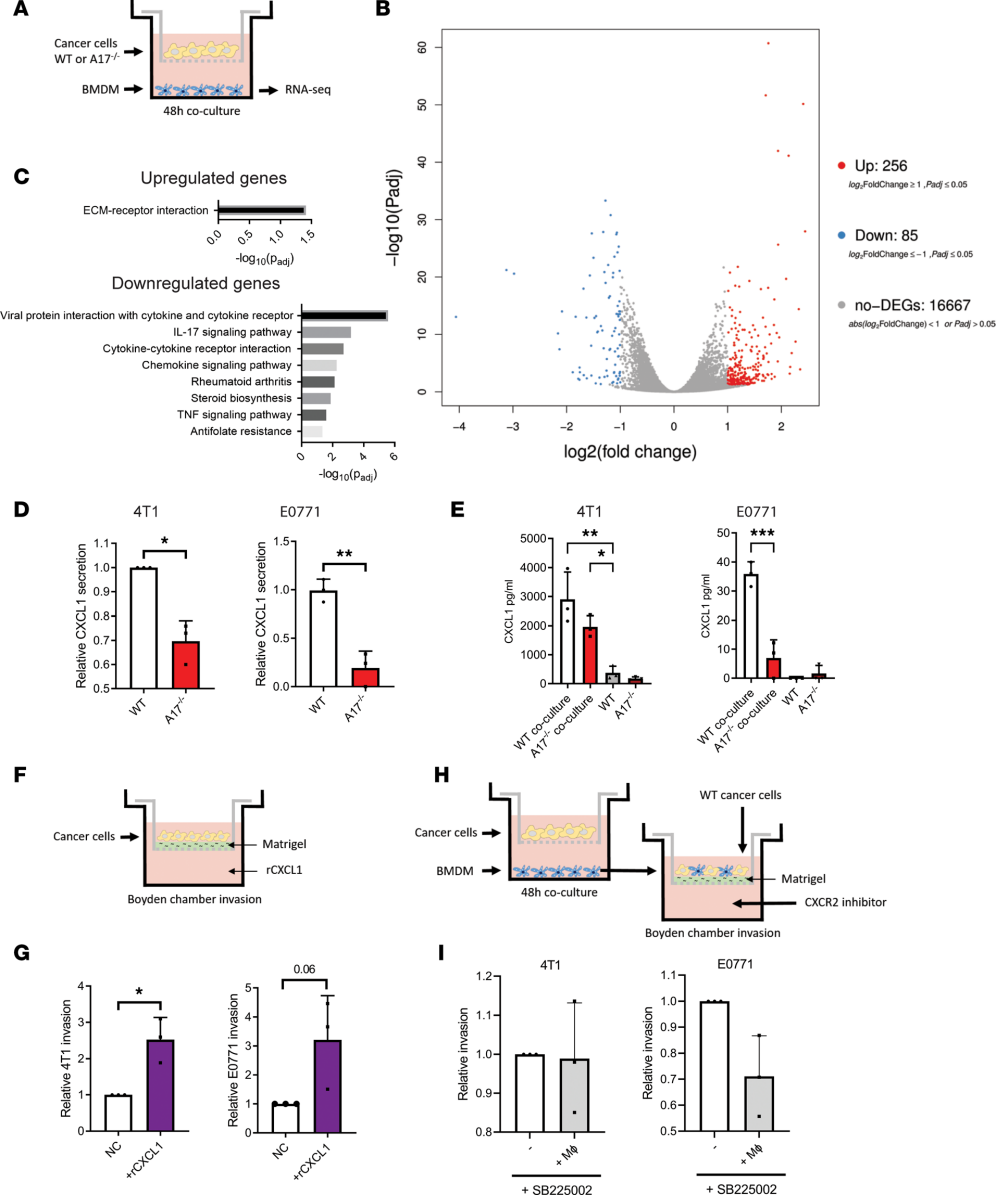
Chemokine secretion from macrophages is responsible for enhanced cell invasion. (**A**) Experimental setup for RNA-Seq of BMDMs upon coculture with cancer cells (**B** and **C**). (**B**) Volcano plot of mRNA transcripts detected by RNA-Seq in BMDMs educated by WT or *Adam17^–/–^* (A17^–/–^) 4T1 cells. Upregulated genes are indicated in red and downregulated genes in blue. DEGs, differentially expressed genes. (**C**) KEGG pathways of significantly upregulated and downregulated genes detected by RNA-Seq, using the g:Profiler tool. (**D**) Relative CXCL1 secretion in WT or *Adam17^–/–^* 4T1 and E0771 cells cocultured with BMDMs and analyzed by ELISA (*n* = 3). (**E**) CXCL1 secretion in WT and *Adam17^–/–^* 4T1 and E0771 cells cocultured with BMDMs or alone, determined by ELISA (*n* = 3). (**F**) Experimental setup of Boyden chamber invasion assays, using solvent (NC) or rCXCL1 as chemoattractant. (**G**) Average number of invaded cells/field of 4T1 and E0771 cell lines (*n* = 3). (**H**) Experimental setup of Boyden chamber invasion assays. (**I**) Relative invasion of 4T1 and E0771 cells cocultured with polarized macrophages and subjected to the CXCR2 inhibitor SB225002 (*n* = 3). Mean and standard deviation indicated. Data in **E** were analyzed by 1-way ANOVA with Dunnett’s multiple comparison test, and data in **D**, **G**, and **I** were analyzed using 2-sided, unpaired Student’s *t* test; *P ≤ 0.05, **P ≤ 0.01, ***P ≤ 0.001.

Macrophages influence cancer cell invasion via multiple mechanisms, including the release of chemotactic growth factors (32), the induction of epithelial-mesenchymal transition (33), and/or the remodeling of the extracellular matrix (34). To get a more in-depth understanding of how ADAM17 influences the macrophage phenotype, we analyzed the transcriptome of cocultured macrophages by RNA-Seq (Figure 6A). In macrophages cocultured with *Adam17*^–/–^ 4T1 cells, we found 256 upregulated genes and 85 downregulated genes, when compared with WT 4T1 cocultured macrophages (Figure 6B and Supplemental Data 2; adjusted *P* ≤ 0.05, fold change ≥ 1). Of the upregulated genes, we found multiple markers associated with an antitumorigenic macrophage phenotype (e.g., *Nos2* and *IL12b*), while of the downregulated genes, we found markers associated with the protumorigenic phenotype (*MRC1* [*CD206*], *CD163*, and *Ch25h*), supporting our qRT-PCR data (Figure 2E). Kyoto Encyclopedia of Genes and Genomes (KEGG) pathway analysis of the up- and downregulated transcripts revealed that genes associated with ECM-receptor interaction were enriched within the upregulated genes, whereas several pathways showed significant enrichment for downregulated genes, including chemokine and cytokine signaling and interaction (Figure 6C).

The specific chemokines downregulated in the cocultured macrophages were *CXCL1*, *CXCL3*, *CXCL5*, *PPBP* (*CXCL7*), *PF4*, *CCL7*, and *CCL12* (Supplemental Data 2). Some of these chemokines contain an N-terminal tripeptide motif glutamate-leucine-arginine (ELR) motif near their N-terminus. ELR-positive chemokines, including CXCL1 and CXCL5, induce cancer cell migration and invasion (35, 36); hence, we tested whether the secretion of CXCL1 and CXCL5 was downregulated in our cancer cell-macrophage cocultures, using the same experimental setup as before (Figure 4A). Using ELISA, we found that the secretion of CXCL1 was downregulated in *Adam17*^–/–^ 4T1 and E0771 cocultures, as compared with the corresponding WT cocultures (Figure 6D). Since CXCL5 was not detected in the E0771 cocultures (data not shown), we excluded this molecule as a common driver of the invasive phenotype in our cell models. Next, we tested whether the cancer cell lines secrete CXCL1 and whether the secretion is influenced by ADAM17. Comparing the secretion of CXCL1 from 4T1 and E0771 cells, alone or in coculture with BMDMs, revealed significantly lower or unmeasurable CXCL1 levels in the cancer cells alone. Furthermore, CXCL1 secretion in the cancer cells was not affected by *Adam17* knockout (Figure 6E), indicating that CXCL1 originates from the macrophages in our cocultures.

We then analyzed whether CXCL1 can induce cancer cell invasion in our cell systems, using Boyden chamber invasion assays (Figure 6F). Indeed, addition of recombinant CXCL1 increased the invasion of both 4T1 and E0771 cancer cells (Figure 6G). Having shown that CXCL1 enhances cancer cell invasion, we asked whether inhibition of the CXCL1 receptor CXCR2 blocked the macrophage-induced invasion. For that, we polarized BMDMs with WT 4T1 or E0771 cancer cells and performed Boyden chamber invasion assays with the polarized BMDMs together with WT 4T1 or E0771 cancer cells, with or without the CXCR2 inhibitor SB225002 added to the medium (Figure 6H). Indeed, we found that CXCR2 inhibition blocked the macrophage-induced cancer cell invasion for both cell lines (Figure 6I).

**Figure 7:**
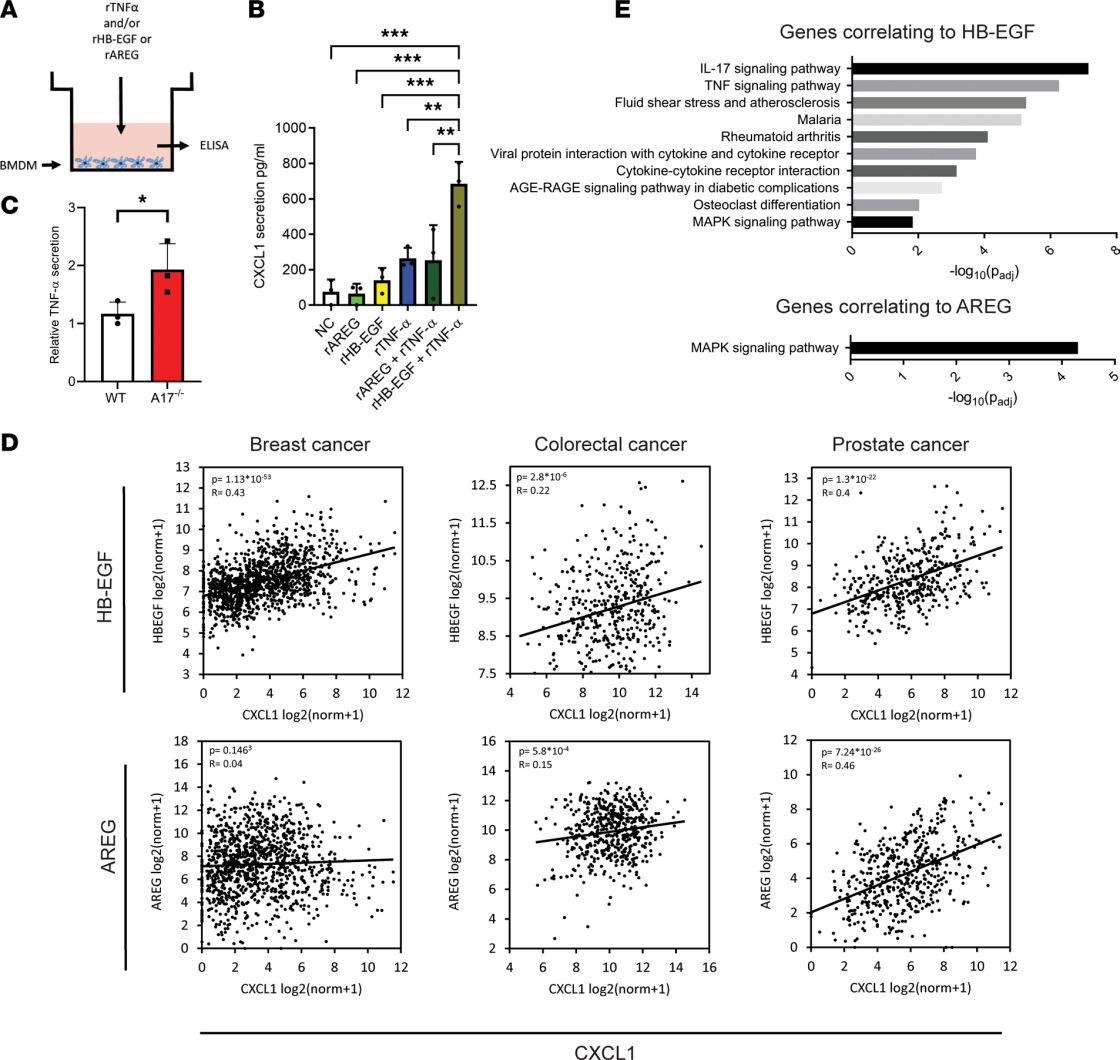
HB-EGF–induced chemokine secretion is responsible for enhanced cell invasion. (**A**) Experimental setup of BMDMs treated with NC, rHB-EGF, rAREG, rTNF-α, or rTNF-α together with rHB-EGF or rAREG. (**B**) Relative CXCL1 secretion by BMDMs upon treatment, determined by ELISA (*n* = 3). (**C**) Relative TNF-α secretion in WT and *Adam17^–/–^* 4T1 cells cocultured with BMDM, determined by targeted MS (*n* = 3). (**D**) Correlations between the expression of HB-EGF or AREG and CXCL1 in breast, colorectal, and prostate cancer, obtained from TCGA database and analyzed using the GEPIA tool. (**E**) KEGG pathways of the top 200 significant genes correlating to HB-EGF or AREG expression in breast cancer from TCGA database and analyzed by the GEPIA tool. Mean and standard deviation indicated. Data in **B** were analyzed by 1-way ANOVA with Dunnett’s multiple comparison test; data in **C** were analyzed using 2-sided, unpaired Student’s *t* test; and data in **D** were analyzed using Pearson’s correlation; *P ≤ 0.05, **P ≤ 0.01, ***P ≤ 0.001.

We then asked specifically whether the decreased release of HB-EGF or AREG could be responsible for the reduced expression and secretion of CXCL1 (Figure 7A). However, treating BMDMs with rHB-EGF or rAREG alone had no effect on CXCL1 secretion (Figure 7B). Also, unlike the effect on macrophage-induced cancer cell invasion, treatment with rCSF-1 alone or in combination with rHB-EGF did not increase the expression of CXCL1 (Supplemental Figure 5). As EGF has previously been shown to act together with TNF-α to increase the secretion of different chemokines (37), we treated BMDMs with recombinant TNF-α (rTNF-α) with or without rHB-EGF or rAREG. As seen in Figure 7B, the combined treatment of rTNF-α and rHB-EGF caused a synergistic increase in CXCL1 secretion, as compared with treatment with either of the 2 factors alone. Interestingly, rAREG treatment did not synergize with rTNF-α, indicating different mechanisms of action between the 2 EGFR ligands. Since MS-based secretome analysis revealed a potentially compensatory increase in secreted TNF-α levels in *Adam17^–/–^* cocultures (Figure 7C), these findings point to HB-EGF as the critical factor. Further supporting the functional link between HB-EGF and CXCL1, we found a positive correlation between the expression of HB-EGF and CXCL1 in several types of human cancer. In line with our experimental findings, there was no correlation between AREG and CXCL1 in breast cancer (Figure 7D).

Finally, to examine whether the deregulated chemokine and cytokine expression in cocultured macrophages correlates to the expression of HB-EGF or AREG in human breast cancer, we analyzed TCGA database for the expression of genes that positively correlate to the expression of HB-EGF and AREG using the GEPIA tool (23). Of the top 200 genes (Supplemental Data 3), we performed KEGG pathway analysis and found for HB-EGF several significantly enriched pathways involving chemokine and cytokine signaling and interaction. However, for AREG, the only significant pathway was the MAPK signaling pathway (Figure 7E).

Together, our data indicate that HB-EGF, shed from cancer cells by ADAM17, amplifies TNF-α and CSF-1 signaling in nearby macrophages. This leads to the secretion of proinvasive chemokines, which promote cancer cell invasion.

## Discussion

Macrophages are diverse in their phenotype and function. They have a high capacity to adapt to external signals, and cancer cells often release factors to hijack macrophages to support tumor progression (38). Hence, blocking these factors to reeducate macrophages toward an antitumorigenic phenotype is considered a promising strategy to stop cancer progression (12). We here identified ADAM17 as a key factor in the education of protumorigenic macrophages. This was documented by a positive correlation between ADAM17 and tumor-supporting macrophages in human patients with cancer and a causative link in mouse tumor models. Mechanistically, we demonstrated that ADAM17-dependent shedding of EGFR ligands directed protumorigenic macrophage education, leading to increased secretion of CXCLs and consequent macrophage-induced cancer cell invasion.

ADAM17 plays an important role during wound healing (39), and its expression in human tumors has been shown to correlate with adverse patient outcome (15, 40). Moreover, we and others have demonstrated a strong cancer-promoting function of ADAM17 activity in murine cancer models (13, 41–43). We now show, in a variety of cancer cell lines, that depletion of ADAM17 alters the macrophage phenotype in human and mouse tumors. Phenotypically, these macrophages are impaired in their ability to induce invasion of cancer cells in vitro, as well as the in vivo cancer cell dissemination in a zebrafish embryo model.

There are currently over 80 confirmed ADAM17 substrates, including the membrane-bound isoform of CSF-1 and multiple EGFR ligands (30). It was previously shown that CSF-1 initiates an EGF-dependent paracrine loop between cancer cells and macrophages that supports cancer cell invasion (32). In our models, however, we found no correlation between ADAM17 depletion and the release of CSF-1, indicating that the majority of extracellular CSF‑1 originates from the soluble isoform (44, 45). Instead, we found that release of the EGFR ligands, HB-EGF and AREG, was reduced in cocultures with ADAM17-deficient cancer cells. We identified the cancer cells as the source of HB-EGF and AREG release, despite the fact that macrophages have been previously shown to release EGFR ligands in other tumor types (46, 47).

Treatment of BMDMs with rHB-EGF or rAREG alone was not sufficient to induce macrophage polarization (i.e., CD206 expression); yet, rHB-EGF has been previously shown to enhance CD206 expression in the less physiologically relevant THP-1 macrophage model (48). We speculate that the EGFR ligands cooperate with signaling factors present in the cocultures to polarize BMDMs. In line with this idea, we observed an increased expression of CD163 when treating BMDMs with rHB-EGF and rCSF-1, whereas treatment with rAREG in combination with rCSF-1 did not alter the expression of CD206 or CD163. The discrepancy between HB-EGF and AREG functions is in agreement with previous findings demonstrating distinct modes of action for the 2 EGFR ligands — e.g., different affinities of the ligands to EGFR (low: AREG vs. high: EGF, TGF-α and HB-EGF) trigger different downstream signaling events (49). Moreover, differences in the actual level of active ligand, distinct kinetics and capacities for homo- versus heterodimer formation, and different downstream effector molecules may contribute to the observed difference in ligand function (50).

Despite the synergistic effect of HB-EGF and CSF-1 on macrophage marker expression, we found no synergistic effects of the 2 factors on the polarization of macrophages toward the invasion-promoting phenotype. Yet, we show that the secretion of both HB-EGF and AREG is crucial for the invasion-supporting phenotype of the macrophages, and we were able to rescue the impaired ability of macrophages educated by *Adam17*^–/–^ cells to induce cancer cell invasion by adding rHB-EGF or rAREG to the cocultures. Interestingly, addition of each of the recombinant EGFR ligands and the individual knockdown fully restored and inhibited, respectively, the invasive phenotype of the macrophages. This could indicate that secreted HB-EGF and AREG act up- or downstream from each other but do not evoke the same signaling in macrophages. Together, our findings identify ADAM17-mediated EGFR ligand shedding as a potentially novel molecular mechanism, used by cancer cells to manipulate and hijack macrophages to support tumor progression.

To get a deeper understanding of ADAM17-dependent macrophage education, we performed RNA-Seq of macrophages following coculture with WT versus *Adam17^–/–^* cancer cells. The RNA-Seq analysis revealed a striking reduction in the expression of multiple chemokines, including CXCL1, which are known to stimulate cancer cell invasion and immune cell recruitment to the tumor (35, 51). Interestingly, there was a substantial overlap between the pathways deregulated in macrophages cocultured with *Adam17*^–/–^ cancer cells and the top 200 genes correlating to HB-EGF in human breast cancer. These mostly included chemokine-associated pathways, thereby suggesting a role of HB-EGF in chemokine signaling. Moreover, we found a positive correlation of HB-EGF and CXCL1 in multiple human tumors, including breast cancer. In line with these findings, it was previously reported that EGFR activation by high-affinity ligands leads to increased chemokine expression and secretion (37, 52). The EGFR ligand TGF-α, for instance, induces the expression of multiple chemokines via increased Akt activation (52). Similarly, EGF is able to induce the mRNA expression of some chemokines, while others were increased by TNF-α and EGF in a synergistic manner (37). Interestingly, TNFR and EGFR both induce NF-κB activation, and the synergism between these factors was mainly seen for chemokines containing a κB binding site in their promoters, such as CXCL1–3 and CXCL8 (53). Likewise, in our work, HB-EGF and AREG alone were not able to induce the secretion of CXCL1 in macrophages, but HB-EGF with TNF-α increased the secretion of CXCL1 in a synergistic manner. Thus, since the secretion of TNF‑α was not decreased upon ADAM17 depletion, these findings point toward HB-EGF as the key factor, inducing the amplifying EGFR signal required to trigger CXCL secretion in our coculture models. It was surprising, however, that AREG with TNF-α did not alter the secretion of CXCL1 in BMDMs, despite previous findings in renal cells, where AREG synergized with TNF-α to induce the secretion of CXCL1, CXCL5, and CCL2 (54). We therefore speculate that the AREG-induced chemokine secretion is dependent on additional tissue-specific factors. This is also supported by the observation that the expression of AREG and CXCL1 did not correlate in breast cancer, while they highly correlated in prostate cancer.

In conclusion, our study uncovers a potentially novel and robust mechanism by which cancer cells regulate the cellular identity of TAMs. We show that ADAM17-dependent shedding of HB-EGF stimulates TAM education and secretion of ELR-positive chemokines, which in turn promote cancer cell invasion. In this way, ADAM17-dependent shedding constitutes a regulatory pathway, which could potentially be targeted to shift the TME from cancer promoting to tumor inhibiting.

Indeed, reprograming macrophages constitutes a promising anticancer strategy, and different approaches have already been developed and shown to boost the antitumor immune repose (12). So far, however, treating cancer with ADAM17 inhibitors failed in clinical trials, mainly due to specificity problems of the inhibitory molecules and lack of knowledge about the function of ADAM17 and other related metalloproteinases in cancer and the TME (55, 56). Given the high number of factors shed by ADAM17, we believe that a more targeted inhibition of specific substrate shedding or corresponding downstream signaling would be a more effective strategy.

It became evident that soluble HB-EGF (sHB-EGF) promotes tumor progression (57), and HB-EGF inhibition has been validated in vitro as a possible therapeutic target in ovarian, breast, bladder, and gastric cancer cells (58). Different sHB-EGF–blocking strategies have been developed to inhibit its binding to EGFR, including monoclonal antibodies (59, 60), a nontoxic mutant of diphtheria toxin (61), the recombinant prodomain of ADAM12 (62), and small inhibitory peptides (63). A first-in-human study with the anti–HB-EGF antibody U3-1565, conducted in a small patient cohort with solid tumors, demonstrated an antitumor response and no dose-limited toxicity (60). In the future, it would be interesting to analyze the effect of U3-1565 on the TME and evaluate whether this treatment could enhance the effect of immune checkpoint inhibitors.

## Methods

### Cell culture.

The cell lines THP-1, SW480, MDA-MB-231 (MDA-231), 4T1, E0771, CT26, and MC38 were purchased from the American Type Culture Collection. The cell lines SW480, MDA-MB-231, and 4T1 were maintained in DMEM containing 100 U/mL penicillin, 100 mg/mL streptomycin, and 10% FBS, and for the cell line E0771, 20% FBS and 10 mM HEPES were supplemented (all from Thermo Fisher Scientific). For the cell line MC38, 0.1 mM nonessential amino acids, 1 mM sodium pyruvate, and 10 mM HEPES (all from Thermo Fisher Scientific) were supplemented. The THP‑1 and CT26 cell lines were cultured in RPMI (Gibco, Thermo Fisher Scientific) containing 100 U/mL penicillin, 100 mg/mL streptomycin, and 10% FBS. THP-1 cells were incubated with 100 nM PMA for 96 hours in RPMI + 10% FBS to generate THP-1–derived macrophages if not described differently. All cells were maintained at 37°C and 5% CO_2_, and exponentially growing cells were harvested when 80% confluence was achieved. The cell lines were regularly tested for mycoplasma infection and authenticated by short tandem repeat profiling (Eurofins).

Primary murine macrophages were generated by flushing the bone marrow from the femur and tibia of the hind legs of 6- to 12-week-old BALB/c or C57BL/6JRj mice (Janvier Labs), followed by incubation for 4–5 days in DMEM containing 100 U/mL penicillin, 100 mg/mL streptomycin, 10% FBS, and 10 ng/mL M-CSF‑1 (PeproTech) in either 6-well (Corning) or 100 mm dishes (Corning) for coculture.

### CRISPR/Cas9 gene editing.

E0771, 4T1, CT26, and MC38 *Adam17*-knockout cells were generated using the CRISPR/Cas9 system as previously described (41). Guide RNAs (gRNAs) were designed using the Wellcome Sanger Institute Genome Editing tool (64) and individually inserted in the vector pSpCas9(BB)-2A-GFP, as described (65). To determine the gRNA editing efficiency, cells were transfected with the pSpCas9(sgRNA)-2A-GFP vectors and verified by Indel Detection by Amplicon Analysis (66). Murine gRNA 5′-ACAAAACTTGAGAGTCGTGG-3′, targeting exon 3, showed the highest editing efficiency and was subsequently transfected into E0771, CT26, and MC38 cells. GFP-positive cells were single-cell-sorted, expanded, and tested by quantitative PCR (qPCR) and Western blot for *ADAM17* knockout. Additionally, we screened positive clones for biallelic frameshifts using Sanger sequencing (Eurofins).

### Expression constructs and cell line generation.

The pcDNA3.1 plasmid containing the murine cDNA of ADAM17 was provided by Stefan Rose-John (Kiel University, Kiel, Germany). ADAM17 inserts were subcloned into the sleeping beauty transposon vector pSBbI-RP (67) and transfected together with the transposase vector pCMV(CAT)T7-SB100 (68) into the 4T1 *Adam17^–/–^* or CT26 *Adam17^–/–^* cells using Fugene HD (Promega), and tdTomato-positive cells were sorted 48 hours after transfection. The expression of ADAM17 was regularly verified by Western blot or qRT-PCR.

### siRNA transfection.

Cells were seeded in 6-well culture plates at a density of 1 × 10^5^ cells per well in 2 mL complete culture medium. After 24 hours, the cells were transfected by using INTERFERin (Polyplus-transfection), or DharmaFECT 1 (Thermo Fisher Scientific) with a siRNA targeting ADAM17 (Origene, M-003453-01-0010), HB-EGF (Origene, SR405442), or AREG (Origene, SR406992) or with a nontargeting siRNA (Thermo Fisher Scientific, SIC001) at a final concentration of 20 nM according to the manufacturer’s instructions. After 48 hours, fresh medium was added to the cells, or the cells were seeded for follow-up experiments.

### qRT-PCR.

Total RNA was extracted from cell lines and macrophages using the RNeasy extraction kit (Qiagen) and treated with DNase I (Qiagen). Quality and concentration of the RNA were measured using NanoDrop (Thermo Fisher Scientific). The RNA was reverse-transcribed using the High-Capacity cDNA Reverse Transcription Kit from Applied Biosystems, Thermo Fisher Scientific. The reverse transcription PCR program was 10 minutes at 25°C followed by 60 minutes at 42°C and 10 minutes at 70°C. qPCR was done using SYBR Green PCR Master Mix from Applied Biosystems Thermo Fisher Scientific. The qPCR program used was 10 minutes at 95°C, followed by 45 cycles of 15 seconds at 95°C, 30 seconds at 55°C–60°C, 30 seconds at 72°C. Primer sequences were mCD163: Fwd 5′-TCCACACGTCCAGAACAGTC-3′, Rev 5′-CCTTGGAAACAGAGACAGGC-3′; mCD206: Fwd 5′-AGAGCCCACAACAACTCCTG-3′, Rev 5′-TCCACTGCTCGTAATCAGCC-3′; mHB-EGF: Fwd 5′-TCTGGCCGCAGTGTTGTCC-3′, Rev 5′-GGTTTGTGGATCCAGTGGGAG-3′; mAREG: Fwd 5′-CTATCTTTGTCTCTGCCATCA-3′, Rev 5′-AGCCTCCTTCTTTCTTCTGTT-3′; mCCL2: Fwd 5′-TTTTGTCACCAAGCTCAAGAGA-3′, Rev 5′-ATTAAGGCATCACAGTCCGAGT-3′; mIL-6: Fwd 5′-CCAGTTGCCTTCTTGGGACT-3′, Rev 5′-GGTCTGTTGGGAGTGGTATCC-3′; mCCR7: Fwd 5′-TCATTGCCGTGGTGGTAGTCTTCA-3′, Rev 5′-ATGTTGAGCTGCTTGCTGGTTTCG-3′; mc-Myc: Fwd 5′-TGACCTAACTCGAGGAGGAGCTGGAATC-3′, Rev 5′-AAGTTTGAGGCAGTTAAAATTATGGCTGAAGC–3′; mNOS2: Fwd 5′-TGTGGCTGTGCTCCATAGTT-3′, Rev 5′-CCAGGGCTCGATCTGGTAGT-3′; mIL-10: Fwd 5′-ATAACTGCACCCACTTCCCAGTC-3′, Rev 5′-CCCAAGTAACCCTTAAAGTCCTGC-3′; mB2M: Fwd 5′-ATTCACCCCCACTGAGACTG-3′, Rev 5′-TGCTATTTCTTTCTGCGTGC-3′; mGAPDH: Fwd 5′-TGTTCCTACCCCCAATGTGT-3′, Rev 5′-TGTGAGGGAGATGCTCAGTG-3′; mTubulin: Fwd 5′-GATCGGTGCTAAGTTCTGGGA-3′, Rev 5′-AGGGACATACTTGCCACCTGT-3′.

### RNA extraction and transcriptome sequencing.

Total RNA was isolated from 60% confluent macrophages growing in a 10 cm dish using the RNeasy extraction kit with subsequent DNase I digestion (both Qiagen), following the manufacturer’s instructions. RNA was quantified using the NanoDrop (Thermo Fisher Scientific), and purity of samples and RNA integrity number (RIN) value were checked on an Agilent Bioanalyzer 2100 system. All samples with an RIN greater than  6 and an OD 260/280 ratio greater than 1.85 were sent to BGI-Copenhagen, Denmark, for transcriptome sequencing, according to BGI standard protocols using BGISEQ-500. All cDNA libraries were sequenced using paired-end strategy (read length 150 bp) on a BGISEQ-500 platform. High-quality reads were aligned to the mouse reference genome (GRCm38, NCBI) with HISAT (69). The expression levels for each gene were normalized to reads per kilobase of transcripts per million mapped reads (RPKM) to facilitate the comparison of transcripts among samples using Bowtie2 (70). A mean log_2_ratio (RPKM of macrophages cocultured with WT 4T1 cells vs. macrophages cocultured with *ADAM17^–/–^* 4T1 cells) of each gene was calculated. Differentially expressed genes were detected using DEseq2 (71) and PoissonDis (72) between the groups. The genes were regarded as differentially expressed when their FDRs were less than 0.001. Further, genes were classified as upregulated when their mean log_2_ratio was above 0.25 or downregulated when their log_2_ratio was below –0.25. KEGG pathway analysis of the up- and downregulated genes was done using the g:Profiler tool (73).

### Western blot.

Whole cell lysates were obtained using RIPA buffer (50 mM Tris-HCl pH 7.5, 150 mM NaCl, 1 mM EDTA, 0.1% SDS, 1% Triton X-100, 0.5% sodium deoxycholate), supplemented with 10 μM Batimastat (MilliporeSigma), 10 mM 1.10 Phenanthroline (MilliporeSigma), and 1× HALT Phosphatase inhibitor (Thermo Fisher Scientific). Protein concentrations were determined by BCA protein assay reagent kit (Thermo Fisher Scientific), and equal amounts of protein were denatured at 95°C for 5 minutes with 4× Laemmli buffer, separated by 10% SDS-PAGE, and blotted onto PVDF membranes (Merck). The membranes were blocked with either 5% milk (MilliporeSigma) or 5% BSA (MilliporeSigma) in TBS-Tween for 1 hours at room temperature and incubated with the primary antibody for 12 hours at 4°C. After washing, the membranes were incubated with horseradish peroxidase–conjugated secondary antibody for 1 hour at room temperature and visualized using ECL detection solution (GE Healthcare) and the ImageQuant LAS 4000 (GE Healthcare). The following antibodies and dilutions were used: rabbit anti-ADAM17 (1:1,000, Abcam, catalog 2051), rabbit anti-EGFR (1:1,000, Cell Signaling Technology, catalog 2232), rabbit anti-pEGFR Y1068 (1:1,000, Cell Signaling Technology, catalog 2234), mouse anti-GAPDH (1:5,000, MilliporeSigma, catalog G8795, clone GAPDH71.1), rabbit anti–β-actin (1:3,000, Cell Signaling Technology, catalog 4067), donkey anti-rabbit–HRP (1:2,000, GE Healthcare, catalog NA934), and sheep anti-mouse–HRP (1:2,000, GE Healthcare, CAT NXA931).

### ELISA.

Quantifications of HB-EGF (R&D Systems, Bio-Techne), AREG (R&D Systems, Bio-Techne), TGF-α (MyBioSource), and CXCL1 (R&D Systems, Bio-Techne) secretion were performed according to the manufacturer’s protocols. For the measurements in cocultures, 2 × 10^5^ cancer cells were seeded 24 hours before coculture in 6-well inserts with a pore size of 0.4 μm (Corning) in 2 mL full medium. The next day, the inserts containing the cancer cells were placed into the wells containing the BMDM. The wells were filled with 2 mL and the inserts with 1.5 mL full medium, and the cultures were incubated for 48 hours. The media from the insert and well were then mixed, centrifuged (1,000*g*, 5 minutes), and stored at –20°C until analysis. To determine the HB-EGF, AREG, and TGF-α secreting cell type, cancer cells and macrophages were separated and incubated for another 16 hours in full medium. For the measurements of CXCL1 in macrophages, 5 days after macrophage isolation, the cells were treated with either of the following: 100 ng/mL HB-EGF (Novus Biologicals), 100 ng/mL AREG (R&D Systems, Bio-Techne), 10 ng/mL TNF-α (R&D Systems, Bio-Techne), or a combination in 700 μL full medium per well in a 6-well plate. PBS containing 0.1% BSA was used as a control. After 24 hours, the medium was centrifuged (1,000*g*, 5 minutes) and stored at –20°C until analysis.

### Conditioned medium for MS-based proteomics.

A total of 22.5 × 10^6^ murine bone marrow–derived cells were seeded at the bottom part of 75 mm Transwell polycarbonate membrane cell culture inserts (Corning) and differentiated toward BMDMs, as described above. For cocultures, 1.5 × 10^6^ 4T1 cancer cells were seeded in 75 mm Transwell polycarbonate membrane coculture inserts (pore size 0.4 μm) and incubated for 24 hours. The next day, inserts containing cancer cells were placed into the wells containing BMDMs in DMEM with 10% FBS, 100 U/mL penicillin, and 100 μg/mL streptomycin and incubated for 12 hours, after which the medium was changed to DMEM without phenol red, serum, and antibiotics (Thermo Fisher Scientific). Following another 12 hours’ incubation, the medium was collected, and the proteolytic activity was inhibited using 0.5 mM PMSF and 10 mM EDTA. Cells and debris were removed by centrifugation (4,000*g*, 30 minutes) and filtering through a 0.22 μm filter. Next, medium was up-concentrated and reconstituted in 50 mM HEPES, pH 7.8, buffer using 3 kDa cutoff Amicon Ultra-15 centrifugal filter units (Merck), following the manufacturer’s instructions. Protein concentration was measured using Pierce BCA Protein assay (Thermo Fisher Scientific) according to the manufacturer’s protocol.

The reference sample was prepared by mixing equal amounts of proteins from each sample. The protein concentration of all samples was adjusted to 10 μg in 90 μL of 50 mM HEPES, pH 7.8, buffer and were denatured at 65°C, reduced with 3.5 mM TCEP, alkylated with 5 mM 2-chloroacetamide, and digested for 24 hours with trypsin (all from MilliporeSigma), using 1:20 trypsin/protein ratio.

### TMT-based proteomics.

Peptides were labeled with TMT 10-plex reagents (Thermo Fisher Scientific) according to the manufacturer’s instructions. For each condition, 10 μg of peptides were labeled at 5:1 TMT/peptide ratio, pooled, and desalted using Sep-Pak C18 columns (Waters). Pooled samples were analyzed on Q Exactive Orbitrap (Thermo Fisher Scientific) coupled with an EASY-nLC 1200 Liquid Chromatography system and a PepMap RSLC C18 50 cm column (2 μm, 100 Å, 75 μm × 50 cm) at 45°C (all from Thermo Fisher Scientific). Data were recorded in a data-dependent acquisition mode over a 140-minute HPLC gradient using full MS scans. MS1 spectra were recorded with 70,000 resolution using 3 × 10^6^ ions, and MS2 spectra were acquired with 35,000 resolution, top 10 precursors with isolation windows of 1.6 *m/z* and 60 ms max injection time, automatic gain control (AGC) target of 1 × 10^6^ ions.

### PRM-based proteomics.

Digested peptides were desalted using custom-made C18 stage tips. Following resuspension in 1% trifluoroacetic acid, 2% acetonitrile containing iRT peptides (Biognosys), 500 ng of peptides per sample were spiked using SpikeMix stable isotope-labeled peptides (JPT) with heavy C-terminal arginine or lysine. All samples were analyzed on the same equipment as TMT-based proteomics in scheduled PRM mode over a 70-minute HPLC gradient. MS1 spectra were recorded with a 60,000 resolution, and MS2 spectra were acquired with a 17,500 resolution, AGC target of 1 × 10^6^ ions and 59 ms injection time.

### MS data processing and statistics.

The TMT raw data file was analyzed as described (74), using ProteomeDiscoverer 2.4 (Thermo Fisher Scientific) against a database compiled from the Uniprot reference proteome for *Mus musculus* (taxid: 10090 v.29-11-2017). Search results were validated using Percolator and filtered to 1% FDR. PRM raw files were searched using ProteomeDiscoverer 2.4 and quantified using Skyline 20.2 (75). For normalization of TMT channels, the function “Total Peptide Amount” was selected in ProteomeDiscoverer 2.4, and the value of each channel was scaled to the channel of the reference sample. Quantified proteins were filtered to master only medium and high FDR confidence. Statistical analysis was performed using CARMAweb (76), applying limma-moderated 2-tailed *t* test and Benjamini-Hochberg *P* value adjustment. Adjusted *P* < 0.05 was considered significant.

For PRM analysis, transition results were exported using Skyline 20.2. They were analyzed using RStudio v.1.2.5001. Between-run technical variation was normalized using summed indigenous and heavy peptides per sample. Next, the spectrum area of each peptide was scaled to average all samples to 100. In each condition, biological triplicates were used, and total sums of indigenous scaled peptides were normalized to the lowest sample per condition. *P* values were calculated using 2-sided Student’s *t* tests; *P* < 0.05 was considered significant.

### Proliferation assay.

The 4T1 and E0771 WT and *ADAM17*^–/–^ cell lines were seeded at 1 × 10^4^ cells per well in 96-well plates in 200 μL full medium. Cells were subsequently incubated at 37°C and 5% CO_2_ in an IncuCyte S3 (Sartorius) for 44 hours, and confluence was recorded every 2 hours. Growth curves were created using the IncuCyte ZOOM software (Sartorius).

### Invasion assay.

Matrigel invasion assays were performed according to the manufacturer’s protocol. Upon rehydration of the precoated Matrigel invasion chambers (pore size 8 μm, Corning), 0.5 × 10^5^ to 2 × 10^5^ DiI-labeled cancer cells were seeded in 500 μL FBS-free DMEM (Gibco, Thermo Fisher Scientific) in the upper chamber of the inserts with or without 10% polarized or treated BMDMs or 50% polarized or IL-4–differentiated THP-1 macrophages. Next, the inserts were transferred into a 24-well plate containing 1 mL full DMEM and incubated for 16–24 hours at 37°C. After incubation, invaded cells were fixed in 4% paraformaldehyde (MilliporeSigma) and visualized under a fluorescence microscope (Zeiss Axiovert 220). Invasion was determined by counting 10 randomly taken pictures at 10× original magnification. For chemokine-induced invasion, 30 ng/mL CXCL1 was added to 1 mL full medium in the lower chamber, and 0.5 × 10^5^ to 2 × 10^5^ cells were seeded in 500 μL FBS-free DMEM in the upper chamber. For the CXCR2 inhibitor experiment, we added 400 nM of the CXCR2 inhibitor SB225002 (MedChemExpress) to the upper and lower chambers. After 16–24 hours’ incubation, invaded cells were fixed in 4% paraformaldehyde (MilliporeSigma), stained with 4% crystal violet (MilliporeSigma), and visualized under a light microscope (Axioplan 2, Zeiss). Invasion was determined by counting the cells in 10 randomly taken pictures (AxioCam, Zeiss) at a 100× original magnification.

### In vivo zebrafish model.

Zebrafish embryos of the strain AB (ZIRC) were raised at 28°C in humidified ambient air. At 24 hours postfertilization, embryos were transferred to aquarium water containing 0.2 mmol/L 1-phenyl-2-thio-urea (PTU, MilliporeSigma) for 24 hours. For macrophage priming, 2 × 10^5^ NC or ADAM17 siRNA–treated SW480 cells were seeded 24 hours before coculture in 6-well inserts with a pore size of 0.4 μm (Corning) in 2 mL full medium. The next day, inserts containing the cancer cells were placed into the wells containing PMA-treated THP-1 macrophages. The wells were filled with 2 mL and the inserts with 1.5 mL full medium and incubated for 48 hours. WT SW480 cells were labeled in vitro with DiO and cocultured THP-1 macrophages with DiI at a concentration of 5 ng/mL in PBS for 1 hour. Subsequently, labeled SW480 cells and THP-1 macrophages were mixed in a ratio of 4:1 and microinjected into the perivitelline space of dechorionated embryos, which were anesthetized with 0.04 mg/mL tricane (MS-22, MilliporeSigma). In the cancer cell–alone group, DiO-labeled SW480 cells were injected in a ratio of 4:1 with DiI-labeled SW480 cells. Between 100 and 300 cells were injected per fish. After injection, embryos with labeled cells in the circulation were excluded, and remaining embryos were transferred to PTU-containing aquarium water and incubated at 34°C in humidified ambient air. After 24 hours, embryos were monitored using a fluorescence stereo microscope (Zeiss stereo lumar with AxioCam MRm, Carl Zeiss). Tumor cell dissemination and macrophage dissemination were determined by counting the green and red cells in the tail region. All zebrafish experiments were approved by the Danish animal experiments inspectorate.

### In vivo mouse models.

Mice were randomly allocated into cages, and mice within the same cage received the same treatment. The injected cell lines were tested negative for murine pathogens by IMPACT testing (IDEXX Laboratories). On the day of injection, 4T1, *Adam17^–/–^* 4T1 (clone 2), and E0771 and *Adam17^–/–^* E0771 (clone 1) cells were harvested, and 1 × 10^5^ (4T1) or 5 × 10^5^ (E0771) cells in 50 μL PBS injected into the fourth mammary fat pad of 7- to 8-week-old female BALB/c (4T1) or C57BL/6JRj (E0771) mice (Janvier Labs). All mice were housed in ventilated cages in groups of 5, maintained in a climate-controlled room at a temperature of 22°C ± 2°C and a relative humidity of 50% ± 5% under a 12-hour light/12-hour dark cycle, and fed a standard diet and water ad libitum. Measurements of the primary tumor size, made using calipers, and the mouse weight were monitored in a blinded fashion 2–3 times a week.

### Patient samples.

The study comprised 159 women diagnosed with unilateral triple-negative breast cancer in the Turku University Hospital, Turku, Finland, between 2000 and 2015. All patients were surgically treated with resection or mastectomy and none of them received neoadjuvant treatment. Complete clinical and follow-up data were collected from patient files available through Auria Biobank, Turku University Hospital, Turku, Finland, and the Finnish Cancer Registry, resulting in an average follow-up period of 8 years. Formalin-fixed (pH 7.0) and paraffin-embedded archival tumor tissue of each patient was available through Auria Biobank. The most representative tumor area of each patient was selected by experienced breast pathologists and was available for the study in tissue microarrays (TMAs). The TMAs were prepared by first identifying representative tumor areas on scanned images of H&E-stained sections (3D HISTOTECH), then punching 1.5 mm thick cylinders from the blocks, and finally, constructing the tissue cores into TMAs using an automated tissue arrayer (TMA Grand Master machine, 3D HISTOTECH).

### IHC.

IHC was performed on paraffin-embedded specimens of the human breast cancer TMA and 4T1 and E0771 mouse tumor sections. After isolation, tumors were formalin-fixed and stored in 70% ethanol at 4°C until paraffinizing. After deparaffinization in xylene and rehydration with decreasing ethanol concentrations, antigen retrieval for the immunostaining was performed using microwave heat–induced retrieval in citrate buffer (pH 6.0, Dako) at 95°C for 10 minutes. Next, endogenous peroxidase activity was blocked using 3% H_2_O_2_ diluted in methanol. Following blocking of nonspecific binding with 5% goat serum in PBS, sections were incubated with anti-ADAM17 rabbit polyclonal antibody (1:2,000, Abcam) or anti-CD163 (1:500; clone EPR19518; abcam) overnight at 4°C. For detection, sections were incubated with working solutions from the VECTASTAIN ABC peroxidase-based kit (Vector Laboratories), following the manufacturer’s instructions. The sections were developed according to the vendor’s protocol for AEC peroxidase substrate kit (Vector Laboratories), counterstained with hematoxylin, and mounted for scoring. TMAs were scored semiquantitatively by 2 independent observers in a blinded fashion. Based on the staining intensity in the cancer cells, TMA sections were scored as negative, weak, moderate, or strong staining. In case of discrepancy in the scoring results, scores were reevaluated and consensus was reached. For statistical analyses, negative- and weak-stained cases were considered as low-expressing group, and moderate and strong staining as high-expressing group. Detection of inflammatory cells expressing CD68 and CD163 was performed using IHC as previously described (24). CD163 infiltration in mouse tumors was determined by counting positive cells/tumor in 3 randomly taken pictures (AxioCam, Zeiss) at a 100× original magnification.

### Data availability.

MS raw files and TMT analysis data are deposited in the ProteomeXchange Consortium (http://proteomecentral.proteomexchange.org) via the PRIDE partner repository (data set identifier PXD030740, ref. 77). RNA-Seq data are deposited in the NCBI Gene Expression Omnibus database (GEO accession GSE212316, GSM6523925, GSM6523927, GSM6523927, GSM6523928, and GSM6523929).

### Statistics.

All statistical analyses were performed using GraphPad Prism version 9.0 or SPSS if not otherwise indicated. The statistical tests used are indicated in the figure legends. *P* < 0.05 was considered statistically significant.

### Study approval.

All animal experiments were performed in accordance with regulations from and with the approval of the Danish Inspectorate for Animal Experimentation, Copenhagen, Denmark. All procedures using human samples were performed in accordance with the ethical standards of institutional and national research committees as approved by the Regional Ethical Review Boards of Turku University Hospital and Auria Biobank, Turku, Finland, and Finnish Cancer Registry, Cancer Society of Finland, Helsinki, Finland (Permit numbers 6/2002, AB15-9859, and TK-53-716-16) and with the 1964 Declaration of Helsinki and its later amendments or comparable ethical standards. The ethical permissions included informed written consent from all individual participants included in the study.

## Author contributions

SPG and MK designed the study. SPG performed, evaluated, and supervised functional assays, Western blots, ELISA, qRT-PCR, zebrafish experiments, mouse experiments, and IHC stainings and scoring; made the CRISPR/Cas9 knockout and overexpression cells; and analyzed the data. LPB contributed to the mouse experiments and performed IHC staining and scoring. KBP performed MS experiments and analyzed the data. MLF performed zebrafish experiments. SS supported MS data analysis. PK collected and provided the triple-negative breast cancer cases. SPG, DHM, UADK, and MK conceived experiments and analyzed the data. SPG and MK wrote the paper.

## Supplementary Material

Supplemental data

Supplemental data set 1

Supplemental data set 2

Supplemental data set 3

## Version 1 08/23/2022

In-Press Preview

## Version 2 09/22/2022

Electronic publication
