# Supplementary material for: ADAM17-mediated EGFR ligand shedding directs macrophage-promoted cancer cell invasion
Source: JCI Insight. 2022 Sep 22;7(18):e155296. doi: 10.1172/jci.insight.155296 (PMC9675555; doi:10.1172/jci.insight.155296)

Supplementary figure 1. Proliferation of WT and *Adam17$^{-/-}$* 4T1 and E0771 cells

A) Relative cell proliferation of WT and A*dam17$^{-/-}$* (A17$^{-/-}$) 4T1 (left) and E0771 (right) cell lines, analyzed over a period of 44h (n=3). Mean and standard deviation indicated. Two-sided, unpaired Student's t-test was applied to test for significant differences.

Supplementary figure 2. Marker expression in macrophages co-cultured with either WT or *Adam17$^{-/-}$* cells

A) Relative IL-10, CCR7, IL6, cMyc, CCl2 and iNOS mRNA expression in macrophages co-cultured with WT or *Adam17$^{-/-}$* (A17$^{-/-}$) 4T1 cells, determined by qRT-PCR (n=4). β_2 microglobulin (B2M) was used as a housekeeping gene. B) Relative IL-10, CCR7, IL6, cMyc and CCl2 mRNA expression in macrophages co-cultured with WT or *Adam17$^{-/-}$* E0771 cells determined by qRT-PCR (n=4). B2M was used as a housekeeping gene. Mean and standard deviation indicated. Two-sided, unpaired Student's t-was applied to test for significant differences: *$p \leq 0.05$, **$p \leq 0.01$.

Supplementary figure 3. Cancer cells educate macrophages towards an invasion promoting phenotype via an ADAM17-dependent soluble factor in several mouse cancer cell lines

A) *Left:* Protein expression of ADAM17 in WT and *Adam17$^{-/-}$* (A17$^{-/-}$) E0771 cell lines, determined by Western blot (representative of 3 repeats). β-actin was used as a loading control. *Right:* Average amount of invaded cells/field of WT E0771 cell lines together with BMDM educated by WT or *Adam17$^{-/-}$* E0771 cells (n=3). B) *Left:* Protein expression of ADAM17 in WT and *Adam17$^{-/-}$* MC38 cell lines, determined by Western blot (representative of 3 repeats). β-actin was used as a loading control. *Right:* Average amount of invaded cells/field of WT MC38 cell lines together with BMDM

educated by WT or *Adam17*$^{-/-}$ MC38 cells (n=3). Data were analyzed by one way ANOVA with Dunnetts multiple comparison test.: *$p \leq 0.05$.

Supplementary figure 4. HB-EGF and AREG siRNA efficiency in 4T1 and E0771 cells

A) HB-EGF and B) AREG knock-down efficiency in 4T1 and E0771 measured by qRT-PCR (n=3). β_2 microglobulin (B2M) was used as a housekeeping gene. Mean and standard deviation indicated. Data in E were analyzed by one way ANOVA with Dunnetts multiple comparison test.

Supplementary figure 5. CXCL1 expression upon CSF-1 and HB-EGF treatment

A) CXCL1 mRNA expression in BMDM upon CSF-1, HB-EGF and CSF-1 and HB-EGF treatment (n=3). Data in E were analyzed by one way ANOVA with Dunnetts multiple comparison test.

Supplementary figure 1

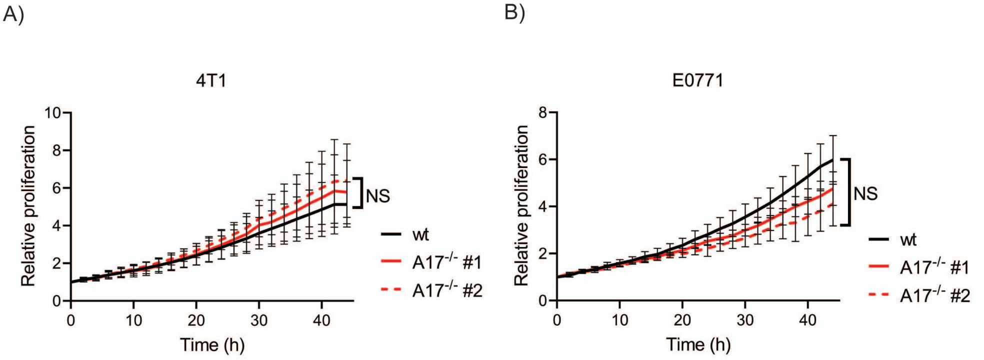

Supplementary figure 2

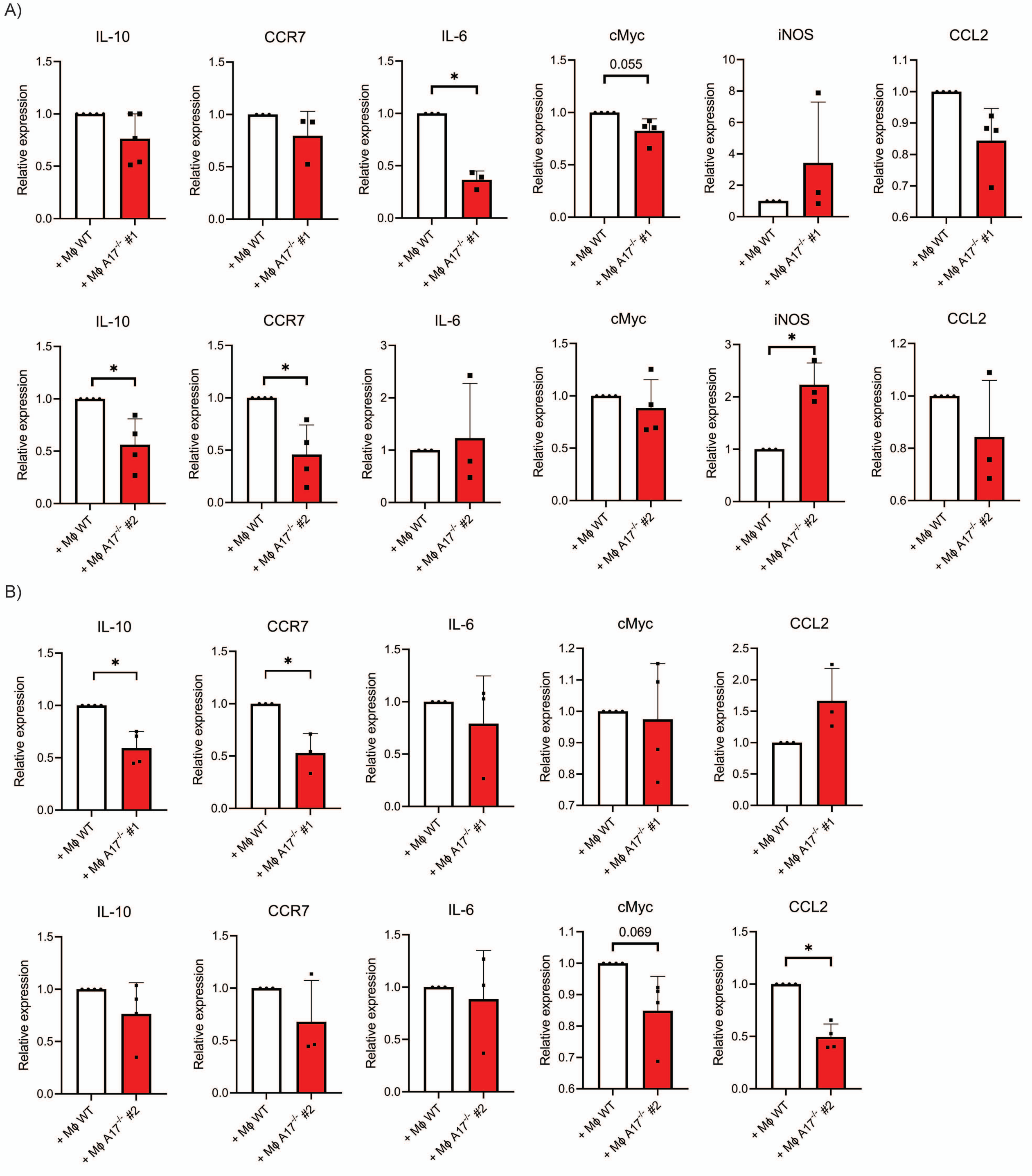

Supplementary figure 3

A)

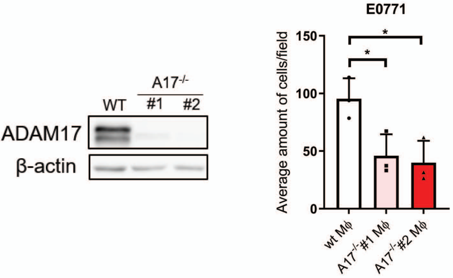

B)

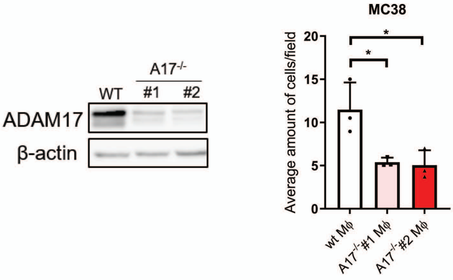

Supplementary figure 4

A)

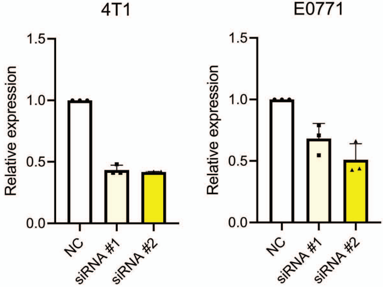

B)

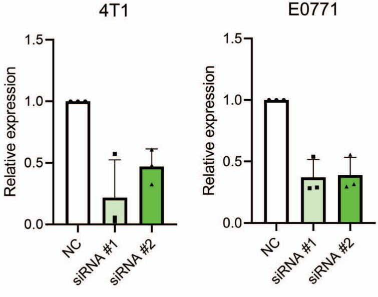

Supplementary figure 5

A)

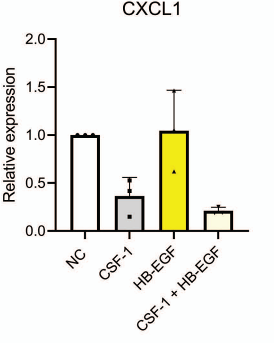

Supplement: Supplemental data [file jciinsight-7-155296-s021.pdf]
